# Receipts

**Published:** 1887-01

**Authors:** 


### RECEIPTS.

It is not usually convenient for us to send receipts for remittances on account of subscriptions by letter mail. They will be enclosed in the next number of the journal sent to the remitter. If, however, any subscriber desires his receipt transmitted at once, he should enclose a stamp for postage. Bills for 1887 will be sent out with the February number.

